# Improving the food environment in Colorado hospitals: a case study

**DOI:** 10.3389/fpubh.2026.1819280

**Published:** 2026-07-16

**Authors:** Reena Oza-Frank, Ynke de Koe, Amy Meyering, Shannon Lawrence, Sharrice White-Cooper, Sahra Kahin

**Affiliations:** 1 Division of Nutrition, Physical Activity, and Obesity, National Center for Chronic Disease Prevention and Health Promotion, Centers for Disease Control and Prevention, Atlanta, GA, United States; 2 Colorado Department of Public Health and Environment, Denver, CO, United States

**Keywords:** food service guidelines, health promotion, hospital, nutrition quality, worksite nutrition

## Abstract

Implementation of Food Service Guidelines (FSG) can improve nutritious offerings and individual health behaviors. This case study demonstrates how a state health department supported hospitals to improve their nutrition environments. The Colorado Healthy Hospital Compact (the Compact) is a statewide initiative designed to implement FSG to improve hospital nutrition environments by increasing the availability of healthier foods and beverages and reducing or eliminating unhealthy items. Developed and implemented by the Colorado Department of Public Health and Environment (CDPHE), hospitals voluntarily participated and completed assessments to monitor changes in the cafeteria nutrition and marketing environments. Of the *n* = 10 participating hospitals that submitted quantitative data in 2021 and 2023, one staff member (eg, food service directors, registered dietitians, wellness coordinators) from 8 of these hospitals completed interviews with CDPHE staff in March 2023 on facilitators and barriers, attitudes and feedback on changes by implementers and clients, maintenance, and experiences with implementation of the Compact. From 2021 to 2023, hospitals showed improvements in meeting standards for healthy sides, entrees, snacks, desserts, and beverages. Hospitals also showed improvements in their pricing strategies and healthy product offerings. The primary barrier to implementation was customer desire for specific foods; facilitators included existing priorities around healthy food environments and buy-in from staff. Common themes of intervention maintenance included having a nutrition policy or contracts, good communication/culture about the Compact, leadership support, and using food service vendor healthy food program(s). The Compact appears to be a feasible approach for implementing healthier nutrition standards in the hospital setting and spreading implementation across hospital settings.

## Introduction

The 2025–2030 Dietary Guidelines for Americans (DGA) recommend increased intake of fruits and vegetables, whole grains, dairy foods, and lean proteins and limited consumption of foods high in sodium, added sugars, and saturated fat (1) to prevent development of diet-attributable diseases (2). The Food Service Guidelines (FSG) for Federal Facilities is intended for government and other types of organizations to operationalize the DGA (based on previous DGAs) into nutrition standards to improve choice, business practices, and support population-level nutrition and health (3). Through simulation models, a recent study showed that implementing FSG in government and private worksite cafeterias could result in significant cost savings related to reductions in cardiovascular disease and mortality (4).

Hospitals can be leaders in reinforcing health recommendations and supporting the health of visitors and staff, thereby supporting the health of the community (5). Health care settings are among the largest employers in the United States (6), and this includes hospitals; thus hospitals have the potential for large and meaningful impact in their communities. Few studies (7), though, have investigated the process and outcomes of FSG implementation in hospital settings.

Using data from 2021 and 2023, we describe the Colorado Healthy Hospital Compact (8, 9) (the Compact), a statewide initiative with approximately 100 hospitals eligible to participate that was designed to implement FSG standards to improve hospital nutrition environments by increasing the availability of healthier foods and beverages and reducing or eliminating unhealthy items.

## Context

### Intervention description

The Compact was developed and implemented by the Colorado Department of Public Health and Environment (CDPHE), which was one of 16 recipients awarded a 5-year cooperative agreement State Physical Activity and Nutrition (SPAN) program from the Centers for Disease Control and Prevention (CDC) Division of Nutrition, Physical Activity, and Obesity (10) to implement FSG. Originally launched on November 14, 2014, the Compact evolved over time to be made up of four programs: healthier food, healthier beverage, marketing (the behavioral design pillar of FSG), and breastfeeding policy and support. This description presents three Compact programs that align with FSG nutrition standards (healthier food, healthier beverage, and marketing). All hospitals across the state were eligible to participate, and if interested in participating, hospitals voluntarily submitted a statement signed by hospital leadership to participate in the program to improve their nutrition environments in cafeterias, vending, and/or patient menus. While CDPHE chose to apply FSG to patient menus, FSG specifically are not intended to address the clinical needs of patients needing specialized medical diets.

### Data collection and methods

Hospitals were asked to complete an assessment of their nutrition environment, at baseline and every 2 years, so CDPHE can monitor (1) changes in the nutrition environment based on FSG and (2) achievements of each hospital in meeting nutrition standards. Collecting and documenting this data also allows hospitals to directly view and monitor their own results and identify areas for improvement. The assessment tool (a downloadable Excel spreadsheet (9)), created by CDPHE, is based on FSG standards, with additional guidelines added by CDPHE to assist with definitions for implementation and following a 2,000 calorie diet (a standard guideline for most adults) (Table 1). Hospitals are asked to transfer aggregate data from the assessment tool to an online survey for data submission to CDPHE.

**Table 1:** Nutrition standards for Colorado healthy hospital compact program in cafeterias, vending, and patient menus, 2021–2023.

	Daily healthy meal	Daily healthy entrée	Healthy sides	Healthy snacks	Desserts	Healthier beverage
On a daily basis	At least one healthy meal in the cafeteria and on the patient menu meets:	At least 40% or ≥60%^a^ of a la carte entrées in the cafeteria and on the patient menu meet the following:	At least 40% or ≥60%^a^ of side dish options in the cafeteria and on the patient menu meet the following:	At least 40% or ≥60%^a^ of snack items in cafeterias, vending, and patient menus meet the following:	Offer 25% of desserts in cafeteria and patient menu that meet:	At least 50% of beverages offered meet the following across cafeteria, vending, and patient menu:
Nutrition requirements	<700 calories Less than 10% of calories from saturated fat* No trans fat Less than 800 mg sodium *Fresh/frozen, non-breaded, non-fried fish cooked in healthy fat is exempt from this rule	<560 calories Less than 10% of calories from saturated fat* No trans fat Less than 600 mg sodium *Soup may be considered an entrée at 8–16 ounces	<250 calories Less than 10% of calories from saturated fat* No trans fat Less than 230 mg sodium *Soup may be considered an entrée at ≤6 ounces	<200 calories* Less than 10% of calories from saturated fat** No trans fat Less than 200 mg sodium Less than 35% of calories from total sugars*** Offer only 2, 1%, or fat free yogurt with no added caloric sweeteners *The following may exceed 200 calories, but must be below 250 calories: plain, unsalted nuts and seeds, fruit and nut mixes, or bars with no added nutritive sweeteners or fats. **The following items may exceed 10% of calories from saturated fat: reduced-fat cheese and part-skim mozzarella; plain, unsalted nuts/seeds, nut/seed butters; fruit nut mixes or bars with no added nutritive sweeteners or fats. ***The following items may exceed 35% calories from total sugar: dried/dehydrated fruit or vegetables with no added nutritive sweeteners; dried whole fruits or pieces with nutritive sweetener for processing or palatability; products containing only exempt dried fruit with nuts/seeds with no added nutritive sweeteners or fats.	<200 calories as a single served item	<40 calories per 8 fluid ounces (excluding 100% fruit juice with no added sugars and unsweetened fat free or low fat [1%] milk)

^a^Meeting at least 40% or ≥60% represents points earned for meeting the standard for the Compact recognition program (platinum, gold, silver, bronze status).

The assessment tool was updated in 2021 to gather more detailed information about food and beverages offered in cafeterias, vending, and patient menus (Table 1). Prior to 2021, the tool only asked yes or no questions on meeting FSG; thus the more detailed data from 2021 were used as the baseline for this case study. Hospitals collected data over a 2 week period or 14 consecutive days, including nutrition data (eg, calories, saturated fat, sodium, trans fats) on various nutrition standards (eg, daily healthy meal, daily healthy children’s meal, daily healthy entrees, healthy sides, healthy snacks, desserts, healthier beverage, and marketing; Tables 1, 2). Hospitals submitted these detailed, nutrient specific data so CDPHE could monitor changes in the nutrition environment based on FSG and achievements in meeting nutrition standards, a more robust approach than relying on hospital self-reported data for meeting nutrition standards. Achievements in meeting standards were used to award points for a recognition program [platinum (81–100 points), gold (66–80 points), silver (41–65 points), bronze (20–40 points)]. Additionally, data from hospitals is included in an interactive online dashboard that displays hospitals participating in the Compact, recognition level achieved, and FSG standards implemented (8). Quantitative data were collected in 2021 and 2023 from *n* = 10 participating hospitals that submitted data in both years. Among the participating hospitals, 70% were acute medical care settings, 80% were located in urban areas, and 50% were medium size hospitals with 451–1750 employees.

**Table 2:** Marketing strategies assessed in cafeterias, vending, and/or patient menus among hospitals participating in the Colorado healthy hospital compact in both 2021 and 2023 (*n* = 10) to promote healthy foods and beverages.

Strategies	Examples
Pricing incentives	Utilize discount or pricing specials on healthier items.
	Offer healthier items at a lower price OR increase the price on non-healthy items.
Marketing with signage or promotions	Utilize signage or displays to highlight healthier items (ie, stoplight or other “healthy icon” symbol).
	Provide calorie and/or nutritional information of all items.
	Prohibit advertising and brand signage of items that do not meet standards (ie, vending or beverage dispenser/machine “wrappers”).
	Offer product samples of healthier items.
Placement and layout of healthy foods/beverages (specific to cafeterias)	Only healthy items displayed at the point of purchase (cash register or check out).
	Offer healthy items as “grab and go.”
Product offerings (specific to cafeterias and patient menu)	Offer half-sandwiches or half-sized entrees.
	Make healthier items default options (fruit instead of chips, salad instead of fries, whole grain bread/bun instead of enriched grain).

We used the quantitative, assessment data of nutrition environments submitted by each hospital to answer the following questions:

- Did the average percent of foods and beverages meeting nutrient standards change between 2021 and 2023?
- How many hospitals increased the number of nutrition standards met between 2021 and 2023?
- How many hospitals increased the number of marketing strategies used to promote the purchase of healthy foods and beverages?

We also collected qualitative data through key informant interviews using an interview guide (Appendix) to answer the following questions about implementation:

- What facilitators did hospitals encounter when implementing the Compact standards?
- Which Compact standards were the most practical and easiest to implement?
- Which Compact standards were the most difficult to implement?
- What was the attitude/culture toward offering more healthy food and beverages within hospitals?
- How will hospitals maintain the changes made over time as a result of their participation in the Compact?
- Overall, what has been the experience for those leading the efforts to implement the Compact standards in their hospitals?

Qualitative data were collected by CDPHE staff through key informant interviews in March–April 2023. Of the *n* = 10 participating hospitals that submitted quantitative data, one staff member (eg, food service directors, registered dietitians, wellness coordinators) from each of *n* = 8 hospitals completed key informant interviews on facilitators and barriers, attitudes and feedback on changes, maintenance, and experiences with implementation of the Compact. Names and position titles were not included in the data analysis.

### Data analysis

Using the submitted hospital assessment tools, CDPHE calculated the percent of sides, entrees, snacks, desserts, and beverages that met nutrient standards in 2021 and 2023 by hospital. The percentages of items by category that met nutrient standards were averaged across hospitals. For example, in 2021, if hospital A had 50% of sides that met the nutrient standards shown in Table 1, and hospital B had 30% of sides that met nutrient standards, then the average percent for healthy sides in 2021 would be shown as 40%.

CDPHE also used the submitted data to determine the percentage of hospitals meeting food and beverage standards in 2021 and 2023. As shown in Table 1, to meet the standard, a hospital needs to meet the minimum threshold of items in that category, only then can the hospital “meet” that standard. For example, for entrees, sides, and snacks, one hospital may have 40% of items that meet the nutrient standard, whereas another hospital may have 100%; both hospitals are counted as meeting the standard.

Similarly, the number and percentage of hospitals reporting use of marketing strategies to promote the purchase of healthy foods and beverages was calculated in 2021 and 2023 to determine improvements in these strategies during this time period. Finally, interview data was inductively analyzed using Dedoose Version 9.0.90 and Microsoft Excel to code and identify major themes.

## Results

### Quantitative results

**Figure 1:**
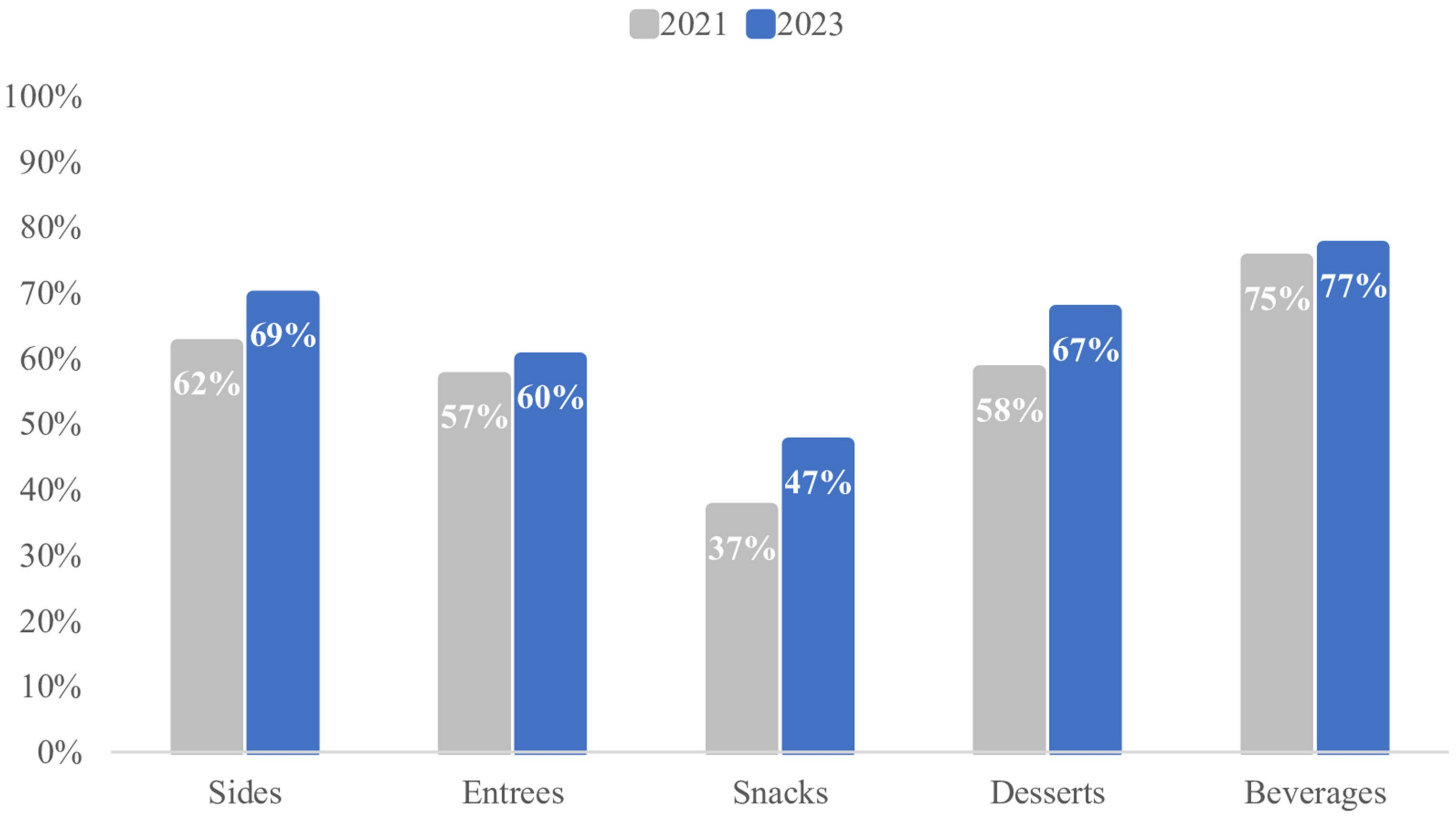
Average percent of foods and beverages offered across hospitals participating in the Colorado healthy hospital Compact (*n* = 10) that met nutrient standards in cafeterias, vending, and patient menus,” 2021 and 2023. “Entrees, sides, and desserts specific to cafeterias and patient menu; snacks specific to vending; beverages across cafeterias, vending, and patient menus. For snacks and desserts, *n*=9 hospitals with available data.

**Figure 2:**
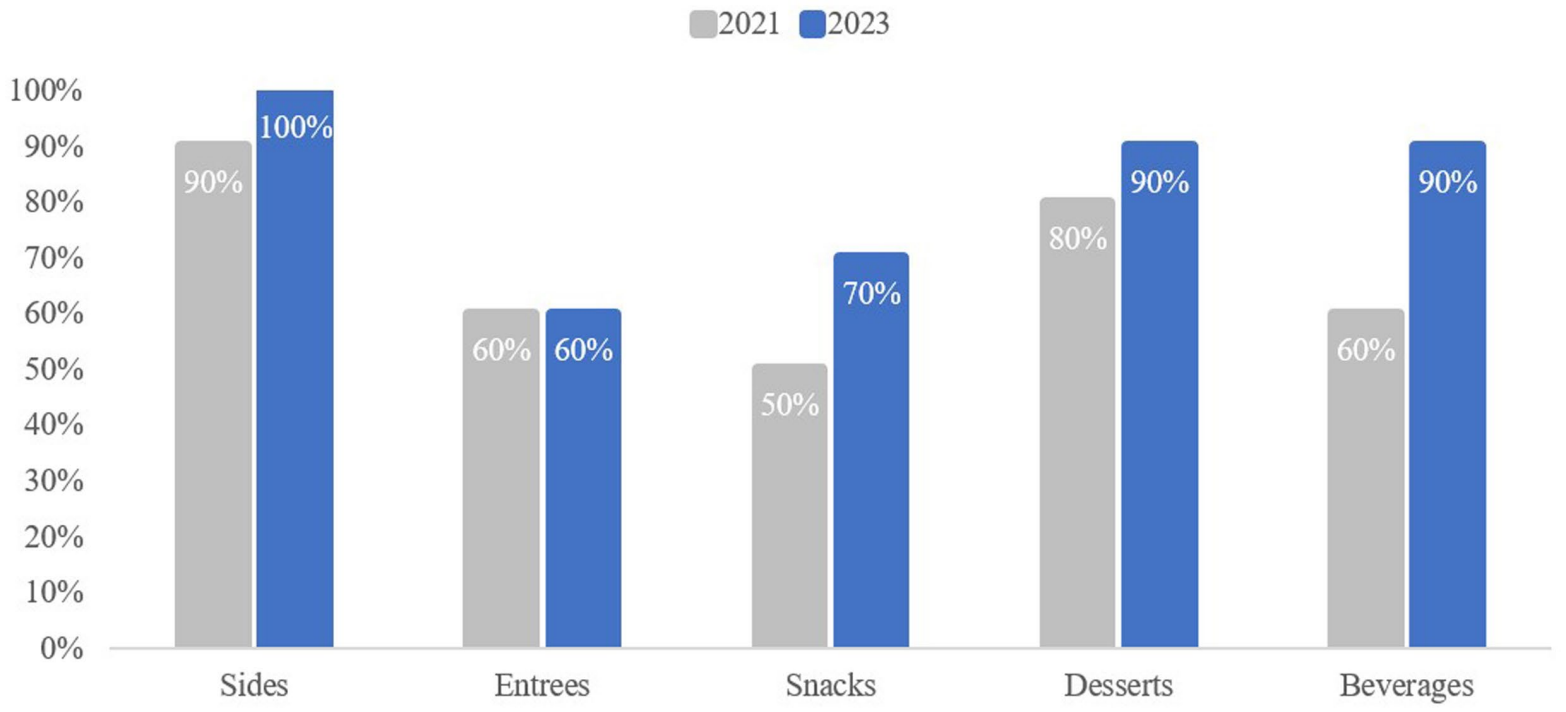
Percent of hospitals participating in the Colorado healthy hospital compact (*n* = 10) meeting healthy food and beverage standards in cafeterias, vending, and patient menus, 2021 and 2023.

From 2021 to 2023, the percent of healthy sides offered increased from 62 to 69%, entrees increased from 57 to 60%, snacks increased from 37 to 47%, desserts increased from 58 to 67%, and healthy beverage offerings increased from 75 to 77% (Figure 1). By 2023, 10/10 of hospitals met the 40% benchmark for healthy sides (up from 9/10 in 2021) (data not shown). Additionally, improvements were made in the percent of hospitals meeting nutrition standards for sides, snacks, desserts, and beverages (Figure 2).

Hospitals showed changes in their healthy marketing strategies from 2021 to 2023. Specifically, more hospitals reported using pricing incentives for healthy items in 2023 (8/10) compared to 2021 (4/10), such as discounting or offering pricing specials on healthier items. One more hospital diversified product offerings, such as offering smaller portions or making healthier items the default option between 2021 and 2023. There were also changes that were not healthier. Specifically, there was no change in the proportion of hospitals marketing healthy choices with signage or promotions (8/10), and there was a decrease in the proportion of hospitals that changed the placement/layout of healthy foods/beverages (8/10 in 2021 vs. 7/10 in 2023). Finally, 2 of the 10 participating hospitals advanced to a higher level of recognition in 2023 compared to 2021.

### Qualitative results

Most of the facilitators for implementing the nutrition standards highlighted the importance of existing priorities around healthy food environments and of gaining buy-in from staff. A quote from one hospital staff member demonstrated how gaining buy-in was hard but worthwhile and appreciated: “I have heard conversationally, when I first started working here about how people disliked not having soda and fried food. But now I hear things such as ‘I am eating better and have lost weight because I do not have access to those unhealthy things.’”

Staff found the easiest or most practical nutrition standards to implement were healthy beverages (*n* = 5 individuals; 63%), due to easy swaps and removal of sugar-sweetened items. One barrier reported by staff from 3 hospitals was that calculating the healthy vs. non-healthy beverages was too time consuming. Staff also reported that a barrier to trying to remove sugar-sweetened beverages was met with pushback due to a high proportion of soda drinkers among their customers.

Staff reported that they struggled most with marketing (50%) and snack (38%) standards. Staff had no issue with simple marketing strategies such as placement of healthy items at eye level, providing calorie and nutritional information, utilizing signage or displays to highlight healthier items, and offering healthy items as “grab and go.” Marketing strategies that were more challenging included prohibiting unhealthy signage and offering product samples of healthy items. Hospitals also identified challenges with finding healthy snacks that fit the exact portion size (eg, smaller size) and nutrition criteria of the nutrition standards.

Some staff reported gathering feedback from their employees and customers about attitudes towards the changes to the nutrition environment. The majority of feedback reported was from employees, and 3 hospitals received a few comments from their community. Feedback from staff and the community was generally positive about the changes (88%). A few hospitals (38%) noted that many staff/customers still wanted comfort foods available and were concerned about too many changes to the food.

Common themes of intervention maintenance mentioned were having a nutrition policy or contracts [50%; (eg, procurement of healthy snacks, restricting or banning sugar-sweetened beverages)], good communication/culture about the Compact (50%), leadership support (38%), and using existing healthy programs available through existing vendors (38%). Staff from all 8 hospitals expressed having positive experiences with leading the efforts of implementing the nutrition standards. Most staff (88%) mentioned that gaining leadership support and buy-in was a key part of their experience with leading the implementation. Half of the staff mentioned the need for internal committees/collaborations with representation from across the hospital.

## Discussion

This case study demonstrated The Compact appears to be a feasible approach for implementing healthier nutrition standards in the hospital setting and spreading implementation across hospitals. Previous studies showed similar qualitative results regarding facilitators of FSG implementation in hospital cafeterias including the need for leadership support and collaborating with others outside of nutrition services for program buy-in and barriers such as customer desire for specific food items (11, 12). Additionally, having nutrition policies can facilitate building changes into contracts/procurement processes to further assist with long-term maintenance of these changes (13).

Initiatives to improve the healthfulness of food served in hospitals in New York City (5) and Philadelphia (14) have also shown improvements in introducing healthy food and beverages. The Compact extends previous work by demonstrating the role a state health department can play in supporting implementation of FSG in hospitals and possibly other worksite settings. CDPHE staff time was used to provide overall program management, facilitate Compact steering committee meetings, create the program assessment tool for data collection and monitoring, review and analyze assessment submissions, manage the recognition process, and provide technical assistance to participating hospitals.

### Limitations

Despite 36 of the 100 hospitals in Colorado having previously submitted data for the Compact, only 10 hospitals were represented in the case study presented here for data consistency across 2021 and 2023 data collection, limiting transferability of the findings within and outside Colorado. Additionally, hospitals that participated in the Compact may have already implemented or been on a path to implement and meet healthy food and beverage standards, which may have contributed to potential volunteer bias. Finally, data was self-reported by hospitals, rather than collected through food audits or sales data. Sales data would have allowed evaluation of sales of affected items, which is important for understanding consumer behavior and for making the business case for FSG (12). Hospitals cited many reasons for not submitting sales data such as time or resources to collect this data separately, or inability to separate data by specific healthy food and beverage items.

### Implications for practice

Through the Compact, Colorado hoped to provide evidence that collaborations between public health and hospitals can successfully change food environments and to demonstrate the usefulness of data-collection tools to help hospitals track, inspect, and improve the healthiness of their food and beverage offerings. The findings revealed key factors that hospitals need in place to succeed in implementing FSGs and improving their nutrition environments such as securing leadership support and buy-in, and finding ways to change attitudes towards healthy foods and beverages (among both staff and customers). Another important consideration for success was increased usage of marketing strategies such as pricing incentives for healthier items. A significant factor in the hospital achievements was the use of the Colorado Healthy Hospital Compact’s multifaceted approach facilitated by CDPHE which included: collecting quality quantitative (9) and qualitative data (Appendix), providing regular support, engaging hospitals through collaborative meetings, recognizing hospitals’ achievements, and sharing successes publicly through a recognition program. The Compact model provides a framework for understanding how hospitals and other worksites can implement and operationalize the FSG, and ultimately make healthier foods and beverages not only accessible, but preferable.

To maintain momentum with the program, hospitals participating in the Compact have transitioned to Health Links®, an employee wellness program, and continue to work on FSG implementation as well as other components of Total Worker Health®. Health Links®, based in the Center for Health, Work & Environment at the Colorado School of Public Health at the University of Colorado Anschutz Campus, is a program that champions health and safety at work by offering evidence-based Healthy Workplace Certification and advice to help organizations and their team members achieve Total Worker Health®. Health Links® will keep the compact branding and levels of recognition.

## Data Availability

The raw data supporting the conclusions of this article will be made available by the authors, without undue reservation.
