# Supplementary material for: Improving the food environment in Colorado hospitals: a case study
Source: Front Public Health. 2026 Jul 16;14:1819280. doi: 10.3389/fpubh.2026.1819280 (PMC13422542; doi:10.3389/fpubh.2026.1819280)
Supplement: Supplementary file 1 [file Data_Sheet_1.pdf]

Colorado Healthy Hospital Compact

Interview Guide - CHHC Evaluation

Interviewee Information

Respondent Name: Hospital Name:

Title of Position: Phone:

Date of Interview: Name of Interviewer:

Introduction/Background

Hello,______(interviewee's name), I'm _________ and this is my colleague _________, who will be taking notes during this call.

I would like to recap the purpose of our call and then we can begin our discussion.

As you may recall, the Colorado Healthy Hospital Compact (or Compact) is funded through a Centers for Disease Control and Prevention (CDC) grant. The Compact is part of a strategy that is looking to increase healthier foods and beverages being offered in worksite settings in an effort to improve the health of employees, visitors and patients. The purpose of this interview is to gain insights into your experiences participating in the Compact, understand your progress implementing the Compact standards, successes and challenges, and to understand sustainability of the changes your hospital has made. This information will be used as a part of the evaluation report we will submit to the CDC on behalf of participating hospitals.

I would like to assure you that all of the information that you share with us in this call today will be kept anonymous. Any direct comments will be scrubbed of individual identifiers including your name, title, hospital and any specific details of your work that could betray your individual identity before being shared. You will have a chance to review your responses and retract or revise statements made before the report is published.

Our call should only take about 30 - 45 minutes, but we have scheduled an hour just to make sure we have enough time. If we get through all of the content early, we will end the interview and you will have some of your time back.

I would like to record this call to help fill in any gaps in our notes. When you set up this appointment [in Calendly], you indicated that a recording is okay with you. **Just to confirm- is this still the case?**

Do you have any questions or concerns before we get started?

[press record button on Zoom]

Compact Implementation, Successes and Challenges

1) Motivation:
    a) [For a long term hospital] What motivated your hospital to continue participating in the Compact?

    b) [For hospital new this grant cycle] What motivated your hospital to participate in the Compact?

2) Overall, what has been your experience leading the efforts to implement the Compact standards in your hospital?

    a) How have you involved other staff or departments in the implementation of Compact standards?

        i) Did you form an internal committee with representation from food service, human resources, lactation, communication, and leadership?
        Prompts,if “yes”:
            (1) Which departments were on this committee?
            (2) How long has this committee been working together on Compact standards?
            (3) What useful or helpful things have come out of having this committee?

        Prompt if “no”:
            (4) Why have you not convened a committee?
                (a) I.e Not aware of creating an internal group? No interest from others?

3) The Compact is made up of four programs: Healthy Food, Healthy Beverages, Marketing, and Breastfeeding.

    a) Which Compact standards were the most practical and easiest for your hospital to implement?
        i) Prompts:
            (1) What made those standards easy or practical?

    b) Which Compact standards were the most difficult for your hospital to implement or that you've avoided implementing?
        i) Prompts:
            (1) What was it about that/those standards that made them difficult?

4) What was your greatest success (or successes) with implementing the Compact standards?

5) What barriers or challenges did your hospital encounter when implementing the Compact standards?
    a) Were or are there any possible resolutions to address these barriers? [If yes], please describe.

Sustainability

6) Is it likely your hospital will maintain/sustain the Compact standards you've implemented so far? Why or why not?
    a) **[IF YES]** How will your hospital sustain the Compact standard changes specifically?

        i) What types of actions, policies, or language in food service vendor contracts will help support sustainability?
    b) [IF NOT] Why are you not able to maintain or sustain changes you have made with Compact standards? What might your hospital need or what would help your hospital to be able to sustain the Compact standards you have implemented so far??

Community and Employee/Patient/Visitor Feedback and Benefit

7) How have your employees, patients, and visitors' attitudes or behaviors toward healthy food and beverages options within your hospital changed over time?
    a) How do you know? Have you collected or looked at any data?

(1) Collecting data such as surveys?
(2) Marketing promotions or sales data?
(3) Anecdotal feedback/conversations?
(4) Other ways?

ii) Prompts, If Yes: please describe the data you collected or reviewed
(1) When did you look at or collect this data?

(2) What did you learn?

iii) Prompts, If not: why haven't you looked or collected any feedback? (Time,interest, need?)

8) How has your community at large benefited by implementing healthy nutritional standards at the hospital?
   a) For example:
      i) Have you done anything to raise awareness in your community about the healthy nutritional changes at your hospital?
      ii) Have you done local food procurement?
      iii) Community gardens?
      iv) Healthy donations to food pantries?
   b) If not or unsure: Do you have any ideas or plans for involving the community in your healthy food efforts?

9) As part of the CDC long-term evaluation, we would like to better understand the purchasing of healthy foods at your hospital and if there have been increases in purchasing or sales of healthy food and beverages across the last 2 years. This type of data can help support and provide evidence of the changes your hospital has made while participating in the Compact.
Some types of data we are looking for are:
   - Point of Sales data from a venue such as a cafeteria or vending machine
   - Purchasing reports, such as what foods and what quantities were purchased for a venue
   - Patient order data

   ○ Data can be for a 1 week period and we would need it for two time points: Spring of 2021 (prior to submitting your first assessment) and Spring of 2023 (when you submit second submission).

   a) Do you collect any of this sales or purchasing data for any of the venues in your hospital?
   b) [If yes] Would you be able to share any of this food service/sales data that you collect with us?
      i) [If Yes] Great! We only need you to pick one standard you want to share data with us about, we can set up another time to discuss exactly what needed.

10) Can you please confirm a few details about your food service provider, suppliers and vending contractors...
    a) From our records, it appears your… Is that correct? **OR**
    b) Who do you use for… ?

**Food Service Provider:** Runs cafeteria operations, staff, etc.	Sodexo Aramark Self-Operated<br>Other, Please List:
**Food Supplier/Distributor:** supplies raw materials and packaged items for food prep	USFoods Sysco Local Farmers<br>Other, Please List:
**Vending Supplier or Contractor:** supplies snack and beverage vending	Canteen Other, Please List:

11) Lastly, did this interview spark any ideas or thoughts that weren't explicitly covered by the questions which you would like us to know?

That is all the questions we have - Thank you for your time and your dedication to the Colorado Healthy Hospital Compact.
(Mention gift cards and will receive them soon)

Interview Guidance - Things to Consider/Prepare

1. Reserve 1 ½ hours for the call on the conference spreadsheet in case you need to continue past the one hour mark.
2. At least one week prior to call, send hospital contact the KII questions (not this doc/we will update a different one with just high-level questions).
3. Prepare for each hospital call by reviewing the database/hospital assessment data - you should know what programs and standards they've implemented/achieved, any food service/vendors they've reported previously (will need to confirm and update if needed).
4. Keep in mind, hospitals may not know/use the Compact language as we do. Prepare other ways to ask the questions if your first question doesn't do the job.
5. Be mindful of how hospitals have answered previous questions so you do not make them repeat themselves. Sometimes you may need to rework a question to probe a bit further about a specific detail OR if they've already answered a question then you may want to skip to the next.
6. Use reflective listening techniques when you don't want someone to repeat the previous idea, but want them to go deeper into a specific thing. (i.e., You mentioned previously about working on garden initiative at the hospital, can you tell me more about your plans or any ways you may involve the community?)
7. Don't move to a new topic prematurely. Don't leave important issues/information hanging - you might run out of time before you can return to them. Also, you will get more useful information by discussing one topic at a time.
8. Don't get stuck on a question. Sometimes you just won't get the information you want from a particular respondent. Know when to move on so you don't frustrate yourself or antagonize your respondent by trying to elicit info that she/he does not have, cannot articulate, or isn't willing share.
9. Don't let the interview go much over an hour; if you are seeing that you need a little more time, ask them if they have the extra time to answer the last few questions or if you can at least ask one last (important) question to wrap things up. The people you chose as key informants from each hospital are likely busy. The quality of conversation can deteriorate if they feel rushed or if you're not respecting their time. These are partners we want to continue to collaborate with. Don't forget to record the interview and take notes!!!
10. Interview Prep: Have notes/info on what hospitals have implemented and not implemented.

11. Practice - read through the entire opening script and questions prior to the first interview. Let us know if questions seem to be landing wrong or don't make sense to the first couple of interviewees so we can make tweaks to questions.
